# Immunomodulatory Role of Microbial Surfactants, with Special Emphasis on Fish

**DOI:** 10.3390/ijms21197004

**Published:** 2020-09-23

**Authors:** Sib Sankar Giri, Hyoun Joong Kim, Sang Guen Kim, Sang Wha Kim, Jun Kwon, Sung Bin Lee, Se Chang Park

**Affiliations:** Laboratory of Aquatic Biomedicine, College of Veterinary Medicine and Research Institute for Veterinary Science, Seoul National University, Seoul 08826, Korea; ssgiri@snu.ac.kr (S.S.G.); hjoong1@nate.com (H.J.K.); imagine5180@gmail.com (S.G.K.); kasey.kim@gmail.com (S.W.K.); kjun1002@naver.com (J.K.); lsbin1129@naver.com (S.B.L.)

**Keywords:** microbial surfactants, glycolipids, lipopeptide, surfactin, anti-inflammatory, fish culture, immune responses

## Abstract

Microbial surfactants (biosurfactants) are a broad category of surface-active biomolecules with multifunctional properties. They self-assemble in aqueous solutions and are adsorbed on various interfaces, causing a decrease in surface tension, as well as interfacial tension, solubilization of hydrophobic compounds, and low critical micellization concentrations. Microbial biosurfactants have been investigated and applied in several fields, including bioremediation, biodegradation, food industry, and cosmetics. Biosurfactants also exhibit anti-microbial, anti-biofilm, anti-cancer, anti-inflammatory, wound healing, and immunomodulatory activities. Recently, it has been reported that biosurfactants can increase the immune responses and disease resistance of fish. Among various microbial surfactants, lipopeptides, glycolipids, and phospholipids are predominantly investigated. This review presents the various immunological activities of biosurfactants, mainly glycolipids and lipopeptides. The applications of biosurfactants in aquaculture, as well as their immunomodulatory activities, that make them novel therapeutic candidates have been also discussed in this review.

## 1. Introduction

Microbial surfactants or biosurfactants (BS) are surface active molecules produced naturally by microorganisms. They comprise both hydrophobic (e.g., hydrocarbon (saturated or unsaturated) chains or fatty acids) and hydrophilic (e.g., acids, peptides, mono-/di-/poly-saccharides) moieties [1]. Due to their amphipathic nature, BS aggregate at interfaces and reduce the interfacial tension, thus increasing the water solubility of hydrophobic compounds. They are less toxic, biodegradable, biocompatible, and stable in a wide range of pH, temperatures, or salinity [1,2]. They also exhibit diverse biological activity and can be produced using various renewable resources [1,3,4,5]. Based on molecular weight, BS are divided into two classes: (a) high-molecular weight BS (bioemulsifiers, such as lipoproteins) and (b) low-molecular weight BS (e.g., glycolipids) [6]. BS are divided into 5 types based on their structure: (a) lipoproteins or lipopeptides (e.g., viscosin, surfactin, subtilisin, polymixin, amphisin, putisolvin, etc.), (b) glycolipids (e.g., cellobiolipids, rhamnolipids, sophorolipids, trehalolipids, etc.), (c) phospholipids, natural lipids, and fatty acids, (d) polymeric BS (liposan, emulsan, mannoprotein, biodispersan, polysaccharide protein complex), and (e) particulate BS [7]. Major BS classes are shown in Table 1. The higher production cost is an important limiting factor for the wide spread use of BS, which can be reduced by utilizing cheap and renewable feedstock, as well as scaling up of synthesis and recovery [8]. It is important to note that there are marked differences between biosurfactants and bioemulsifiers. Although both can efficiently emulsify two immiscible liquids, such as hydrocarbons or other hydrophobic substrates, bioemulsifiers are not effective in reducing surface tension. Bioemulsifiers are said to possess only emulsifying activity and not surface activity [9]. Therefore, all biosurfactants are also bioemulsifiers by definition, but all biomulsifiers are not biosurfactants. 

The global surface-active agents (SAA) market is growing steadily at an annual average growth rate of 6.75% and is expected to expand to an USD 3.21 billion market by 2025 [10]. Recently, there has been increased attention on microbial surfactants. BS are suitable for industrial applications as they exhibit anti-adhesive, anti-biofilm, and antimicrobial activities. BS have been exploited for applications in gene transfection, vaccine delivery, immunomodulation, cancer therapy, etc. [4,10,11,12,13,14,15]. BS have been used as immunostimulants in fish culture and have been explored to improve the defense mechanisms of fish against various diseases. Due to the diverse effects of BS on immune responses, the current review focused on the latest advances in the BS-driven immune responses in fish. 

## 2. Effect of BS on the Immune System

**Figure 1:**
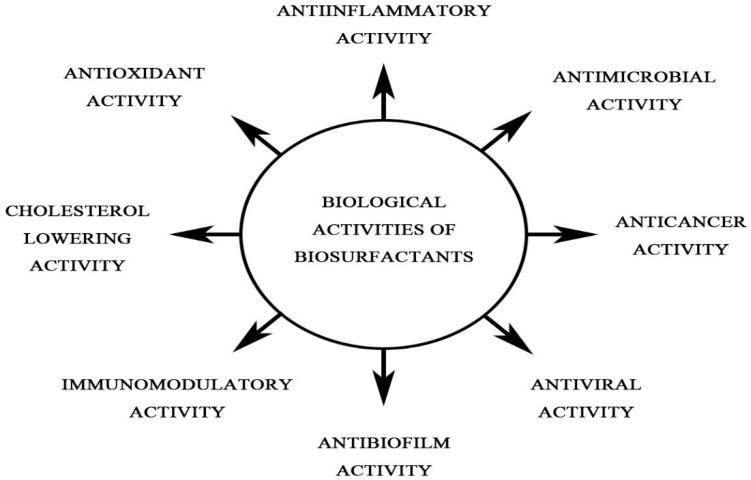
Biological activities of biosurfactants.

Several BS are known to modulate immune responses both at cellular and humoral levels (Figure 1). In medicine, BS are being explored in areas, such as antibacterial therapy, antiviral therapy, gene and drug delivery, and immunomodulation [16]. BS isolated from various microorganisms exhibited antimicrobial properties against a broad range of opportunistic pathogens, including multidrug resistant strains of *Escherichia coli*, *Acinetobacter baumannii*, and *Staphylococcus aureus* [17,18]. The effects of important BS (e.g., glycolipids, lipopeptides, etc.) on the immune responses are discussed below.

### 2.1. Glycolipids

Glycolipids consist of carbohydrate moieties linked to fatty acids. Based on the nature of the carbohydrate moiety, glycolipids are categorized into rhamnolipids, sophorolipids, trehalolipids cellobiolipids, lipomannans, galactosyl-diglycerides, mannosylerythritol lipids, lipomannosyl-mannitols, diglycosyl diglycerides, and monoacylglycerols [19]. The most common glycolipids (trehalolipids, rhamnolipids, and sophorolipids) are produced by *Rhodococcus*, *Pseudomonas* sp., and yeast strains, respectively. 

Rhamnolipids are known for their antiviral, cytotoxic, hemolytic, antibiofilm, antimicrobial, antiadhesive, and algicidal properties [11,14,20]. Rhamnolipids interfered with the internalization process of macrophages, thereby inhibiting their phagocytic abilities [21]. It has been reported that the presence of rhamnolipids causes macrophage lysis and necrosis of polymorphonuclear leucocytes (PMNs) [22]. Sana et al. [23] showed that rhamnolipid interacts with the nonpolar part of the cell membrane of *E. coli* and *S. aureus*. The membrane disintegrates leading to the penetration of the cell wall and plasma membrane by pore formation and subsequent leakage of inner cytoplasmic materials leading to cell death. Further, rhamnolipid inserts its shorter acyl tails into the cell membrane and attacks the configuration of the cell wall and plasma membrane [24]. Sophorolipids possess antibacterial, antiviral, anti-cancer, and anti-mycoplasma activities [24,25,26]. In an experiment, Bluth et al. [27] induced intra-abdominal sepsis in rats and then treated them with sophorolipids; this increased the survival of rats and blocked the detrimental effect of septic shock by attenuating the production of pro-inflammatory cytokines and nitric oxide [27]. Sophorolipids downregulated the expression of *TLR2*, *PAX5*, *STAT3*, and *IL6* in U266 cell lines; thereby reducing the production of IgE [27]. A complex BS synthesized by *Rhodococcus ruber* exhibited immunomodulatory properties [28]. The glycolipid BS complex had no considerable effect on the proliferative action of peripheral blood leucocytes. However, it activated the production of proinflammatory cytokines (IL-1β and TNF-α) without affecting the in vitro production of IL-6 in the monocyte fraction. In the mononuclear fraction, the glycolipid BS did not affect the production of either of those cytokines [29]. These results demonstrated the potential of glycolipid BS for immunomodulatory and antitumor activities.

Trehalose dimycolate (TDM), trehalose monomycolate (TMM), and trehalose tetraesters are glycolipids produced by various bacteria, and these molecules possess diverse immunological activities. TDM-coated charcoal particles and TDM-loaded poly-DL-lactide-coglycolide microspheres induced strong inflammatory responses in mice [30]. Levels of cytokines (IL-4, IL-6, IL-10, IL-12, TNF-α, and IFN-γ) were higher in the mice lung cells, and elevated nitric oxide (NO) production was observed in the culture supernatants of bronchoalveolar lavage cells. The study suggested that TDM might trigger the production of cytokines, which might contribute to the persistence of infection. These findings are critical for better understanding of the immunostimulatory activities of TDM [30]. Trehalose BS produced by *R. ruber* IEGM231 stimulated the production of reactive oxygen species (ROS) and IL-8 in peripheral blood neutrophil cultures. The neutrophil-driven production of IL-1β and TNF-α was not significantly affected by the presence of BS [31]. Crude or purified TDM or TMM from *R. equi* markedly increased the *IFN-γ* transcription in equine peripheral blood mononuclear cells [32]. Chereshnev et al. [33] demonstrated that glycolipid BS complex from *R. ruber* IEGM 231 stimulated the production of IL-12, IL-18, and ROS by the innate immune cells. However, the glycolipid BS complex had minimal effect on the secretion of IL-10 by mononuclear cells and monocytes. Interestingly, glycolipid from *R. ruber* stimulated the cytokine production only when applied as an ultrasonic o/w emulsion [34]. *R. ruber* IEGM 231-derived glycolipid BS inhibited specific and non-specific immune parameters in male albino mice. Striking suppression in the bactericidal potential and production of antibodies and proinflammatory cytokines by peritoneal macrophages was also demonstrated [35]. These results contradicted the observations of a previous in vitro study by the same authors where immunomodulatory activities of BS towards the immunocompetent cell cultures were detected. Therefore, cellular environment might play an important role in the elicitation of immune responses under the effect of bacterial glycolipids. 

Mannosylerythritol lipids (MELs) are a class of compounds with amphiphilic properties, classified as a biosurfactant [15]. Extracellular glycolipid MELs produced by *Candida antarctica* induced apoptosis, growth arrest, and the differentiation of mouse malignant melanoma B16 cells [36]. MELs at a concentration 10 µM or higher caused apoptosis, which was indicated by DNA fragmentation, condensation of chromatin, and sub-G1 arrest. It was found that growth inhibition and apoptosis are closely related to sub-G1 arrest [36]. Six variants of sophorolipids were purified from *Starmerella bombicola* CGMCC1576 [37]. The inhibitory mechanism of a sophorolipid on HeLa cells was investigated. Apoptosis and blockage of cell cycle was observed at G0 phase. Induction of C/EBP homologous protein (CHOP) and *Bip*/*GRP78* gene expression was observed, and caspase-3 and caspase-12 were activated. However, concentration of cytosolic cytochrome C and mitochondrial membrane potential were unaltered. A dose of 500 mg/kg lactonic sophorolipids exhibited 52.06% inhibition without significant toxicity to the tumor-bearing mice [37]. These results suggested the potential of sophorolipids in the treatment of human cervical cancer. 

Sophorolipids (SLs) formed an assembly with commercial bovine lactoferrin (CbLf) and enhanced the absorption in model skin [38]. In human dermal fibroblasts (HDFn), the uptake and post-internalization localization of CbLf, bovine Lf (bLf), and human Lf (hLf) were investigated with or without forming assemblies with SLs using ^125^I-labeled Lfs and confocal microscopy. The HDFn internalized all the 3 Lfs. Transcriptomic analysis indicated that CbLf may be involved in accelerating wound healing, protection of skin from oxidative stress, and immunostimulatory activities. SLs alone modified signaling pathways associated with lipid metabolism and synthesis of vitamins [38]. Therefore, CbLf may exhibit beneficial effects on skin involving the immunomodulatory effect of SLs.

Sophorolipid molecules with different structures obtained from yeast broth were evaluated for anticancer activities on human esophageal cancer cell lines [23,24]. The inhibitory activity of diacetylated lactonic sophorolipid on two cancer cell lines was stronger than that of the monoacetylated lactonic sophorolipid. SLs with one double bond in the fatty acid moiety had robust cytotoxic effect on the esophageal cancer cells, and the inhibition concentration was 30 µg/mL. Thus, anticancer activities of SLs are linked to their structures, namely with higher acetylation degree of sophorose, less unsaturation degree of hydroxyl fatty acid, and lactonization, may be associated with enhanced performance [24]. Yuewen et al. [39] investigated the effects of lactonic diacetyl-SLs (L-SLs) on cancer cells by determining the optimal load rate, slow-release ability test, and cell toxicity. They demonstrated that SLs exert anticancer activity against HeLa cells, and loading on Nanohydroxyapatite increased the slow-release ability of L-SLs.

Ribeiro et al. [40] biosynthesized SLs from *Starmerella bombicola* in a culture medium supplemented with borage oil. Among the SLs, C18:1 lactonic SL in specific concentrations inhibited the migration of MDA-MB-231 cells without compromising cell viability and increased intracellular ROS. Recently, Kristoffersen et al. [41] isolated Rha-C10-C10, Rha-C10-C12, and Rha-C14-C10 monorhamnolipids from *Pseudomonas* sp. M10B774. They demonstrated the cytotoxic effect of the rhamnolipid BS on A2058, HT-29, and MCF-7 cancer cell lines.

### 2.2. Lipopeptides

Lipopeptides are low molecular weight amphiphilic molecules which include one or more lipid chains linked with a peptide head group [42]. Members of the genera *Aspergillus*, *Bacillus*, *Pseudomonas*, *Streptomyces* are main producers of the lipopeptide BS. The genus *Bacillus* produces BS molecules viz. fengycin, iturin, and surfactin. These families share a β-hydroxy fatty acid or cyclic β-amino acid attached to the lipid tail [43]. The biological activities of these compounds may differ based on the cyclisation of the peptide, the type of amino-acid residues, and the length and branching of the fatty acid chain [44]. BS produced by various *Lactobacillus* strains demonstrated a considerable antimicrobial activity against *S. aureus*, *Streptococcus agalactiae*, and *P. aeruginosa* [17]. Surfactin and rhamnolipids isolated from *Bacillus amyloliquefaciens* and *P. aeruginosa* exhibited antimicrobial activity against drug-resistant *E. coli*, *S. aureus*, and pathogenic *C. albicans* [18]. Janek et al. [45] demonstrated that pseudofactin II (BS) interacts with bovine serum albumin (BSA) via a static quenching mechanism. This study will assist in the better understanding of the binding mechanism of pseudofactin to proteins and conformational changes.

The anticancer activity of surfactin, including anti-proliferative and apoptotic effects has been studied on a variety of cell lines [46]. Surfactin has been found to display an antiproliferative effect via cell cycle arrest, apoptosis induction, and survival signaling suppression [24]. Surfactin-mediated cytotoxicity on various cancer models are discussed here briefly.

- Breast cancer: Anticancer activity of surfactin has been studied widely on breast cancer cell lines. *B. subtilis* CSY191 derived surfactin inhibited the MCF-7 breast cell lines with IC_50_ of 9.65 µM at 24 h [47]. Surfactin from *B. subtilis* 573 inhibited the T47D cells in a dose-dependent manner, and 48 h IC_50_ was calculated to be 193 µM [48]. Surfactin has been found to induce apoptosis via the ROS/c-Jun N-terminal (JNK)-mediated pathway. *B. subtilis natto* TK-1 purified surfactin (29 µM) caused 50% viability inhibition, whereas 68 µM surfactin caused 80% inhibition on the MCF-7 cells [49].
- Colon cancer: Studies on surfactin mediated anti-colon cancer activity are limited. Surfactin strongly inhibited the growth of LoVo colon cancer cells, with the 48 h IC_50_ being 26 µM [50]. The anti-proliferative action was mediated by morphological changes, DNA fragmentation, cell cycle regulatory proteins and altered levels of apoptosis. In another study, Sivapathasekharan et al. [51] demonstrated that surfactin derived from *B. circulans* DMS-2 induced moderate toxicity in the HCT-15 and HT-29 colon cancer cells, with the IC_50_ of 24 h being 77 and 116 µM, respectively.
- Leukemia: *B. subtilis natto* T-2 derived surfactin exhibited toxicity in human K562 leukemia cells at different concentrations (2–62 μM) for 24–48 h. Surfactin inhibited the growth of K562 cells in the 24–48 h treatment period at IC_50_ values ranging between 10–20 μM [52].
- Hepatocellular carcinoma: The cytotoxic effect of surfactin on hepatocellular carcinoma was investigated and surfactin-like lipopeptides were found to strongly inhibit the cell viability of human Bel-7402 hepatoma cells, with IC_50_ of 35 ± 12 μM [53]. Wang et al. [54] found that surfactin induced apoptosis in the HepG2 cells via the ROS/ERS/Ca_2+_-mediated ERK pathway.
- Cervical cancer: Lui et al. [53] investigated the effect of surfactin on cervical cancer in HeLa cell line. The IC_50_ dose of surfactin at 16–48 h was ranged between 86.9–50.2 µM [53].

Surfactin reduced the production of proinflammatory cytokines in macrophages stimulated with lipopolysaccharides [55]. Surfactin attenuated the NF-κB activation via blocking the IKK-β degradation and reducing the expression of IL-6, iNOS, and IFN-γ [55]. Moreover, Park et al. [56] demonstrated the anti-inflammatory and neuroprotective potential of surfactin on the BV-2 microglial cells stimulated with lipoteichoic acids. Surfactin treatment attenuated the activation of NF-κΒ and activated the cyclic adenosine 3′, 5′-monophosphate (cAMP)-protein kinase A (PKA)-cAMP response element-binding protein (CREB) pathway [56]. Gan et al. [57] explored the mechanisms underlying the adjuvant properties of surfactin. They demonstrated that surfactin acts as non-pathogen-associated molecular patterns. It can modulate the innate immunity of the host via various signaling pathways, including activation of NF-κB and MAPKs, induction of mitochondria-dependent ROS, and induce cell apoptosis to release endogenous danger signals to activate the inflammasomes.

Compounds, including 13-docosenamide, (Z); mannosamine, 9- and *N*,*N*,*N*′,*N*′-tetramethyl, were found in lipopeptide BS isolated from the halophilic *Bacillus* sp. BS3 [58]. BS3 BS exhibited anticancer activity on the mammary epithelial carcinoma cells at the concentrations (0.00025, 0.0025, 0.025, 0.25, and 2.5 µg), and 0.25 µg concentration suppressed the cells up to 24.8%. Lipopeptides stimulated the maturation of dendritic cells via TLR2 signaling [59] and lipopeptide stimulation enhanced the expression of CD54, CD58, CD80, CD83, CD86, and MHC class II molecules, as well as decreased CD32 expression and endolytic activity [59].

Surfactin treatment converted immature dendritic cells (DCs) into functional DCs [60]. This phenomenon increased the expression of MHC-II molecules and other co-stimulatory factors. As surfactin treatment led to increased levels of p65 and decreased the expression of IκB-α, it was implied that NF-κB pathway may be involved. WH1 fungin, a surfactin produced by *B. amyloliquefaciens*, has been found to provoke cellular and humoral immune responses to ovalbumin and Hepatitis-B surface antigens [61]. WH1 induced the production of ROS and enhanced the production of cell surface markers and cytokines. A surfactin isolated from *B. subtilis* TD7 broth exhibited low toxicity on erythrocytes, indicating its potential for biological applications [62].

The molecular structure of lipopeptides influences its biological activities. The antifungal activity of iturinic lipopeptides is affected by the length of their carbon-chains. The elongated fatty acid chain interacts with the cell membrane due to the hydrophobic nature of the long fatty acids [63]. The C_16_ isoform of iturin A from marine *B. megaterium* suppressed the growth of tumor cells by disrupting the Akt pathway leading to apoptosis [64]. In addition, iturin A re-sensitized the docetaxel resistant MDA-MB-231 and MDA-MB-468 breast cancer cells by reducing phosphorylated-Akt expression levels, which led to the inactivation of the Akt pathway [65]. The longer fatty acid chains of these BS might contribute to the increase in the surface-activity of these molecules.

In a recent study, poly(lactic-co-glycolic acid) nanocapsules containing amphiphilic SLs were formulated and evaluated for anti-carcinoma activity [66]. The formulations with 10% poly(ethylene glycol) density achieved more than 80% reduction in cancer cell viability after 72 h and enhanced cellular uptake in CT26 cells. Moreover, animals treated with SL-loaded nanocapsules exhibited 57% inhibition of tumor growth. Thus, hydrophilic poly(lactic-co-glycolic acid) nanocapsules loaded with SLs can address the poor intracellular delivery linked with SLs and may be effective in treating colon neoplasia [66]. Lipopeptides produced by *Acinetobacter junii* showed IC_50_ of 7.8 ± 0.4 mg/mL, 2.4 ± 0.5 mg/mL, and 5.7 ± 0.1 mg/mL against U87, KB, and HUVEC cell lines, respectively [67].

*B. subtilis*-derived iturin A induced both paraptosis and apoptosis in heterogeneous human epithelial colorectal adenocarcinoma (Caco-2) cells [68]. Iturin concurrently induced autophagy in Caco-2 cells treated at an early stage but inhibited autophagy at the later stages. They showed that upregulated expression of p62 was linked to the inhibition of autophagy at the later stages, and iturin-driven anti-tumor activity was executed via multiple pathways [68]. Lipopeptides kill cancer cells via various mechanisms. Lipopeptides can integrate between the semi-permeable membrane(s) of the tumor cells, thus changing the membrane potential and causing cell death [69]. Moreover, lipopeptides inhibit the cancer cell proliferation by inducing apoptosis and cell-cycle arrest. Iturin A inhibited the proliferation of MCF-7 cancer cells via the Akt signaling pathway [70]. Lipopeptides can suppress many signals, such as ERK and PI3K/Akt pathways. The ability of biosurfactants to disrupt cell membranes, leading to a sequence of events that include lysis, enhanced membrane permeability, and metabolite leakage, has also been suggested as a probable mechanism of antitumor activity [71].Although several studies focused on the antitumor/anticancer potential of biosurfactants, further studies are necessary to elucidate the molecular mechanisms involved in such activity.

### 2.3. Immunomodulatory Activity of Other Microbial Surfactants

Some bacteria secrete exopolysaccharides (EPS) which are secreted polymers of sugars. Some EPS are amphiphilic in nature; particularly, BS increases the bioavailability of hydrophobic substrates [63]. The EPS from various lactic acid bacteria (LAB) have been found to modulate immune responses in the host. Hidalgo-Cantabrana et al. [72] suggested that EPS of small molecular weight and/or negative charges are strong immunomodulators. EPS derived from mutant *Weissella confusa* increased the production of IgM and IgG in swiss albino mice, whereas IgA production was increased in the wild type [73]. Both type of EPS had immunostimulatory activity on treat mice [73]. The EPS produced by *Lactobacillus pentosus* LZ-R-17 exhibited noteworthy immunomodulatory activity via enhanced viability of RAW264.7 macrophage cells and increased phagocytosis, activation of macrophages, and stimulation of the secretion of NO, IL-1β, IL6, TNF-α, and IL-10 [74].

EPS of probiotic origin exhibited anticancer activity, and the diversity of sugar composition was found to be responsible for the anti-proliferative activities. EPS from probiotic *Pediococcus pentosaceus* M41 exhibited anticancer activity in Caco-2 and MCF-7 cells [75]. *L. plantarum* NCU116 derived EPS enhanced the expression of pro-apoptotic genes (*Fas, FAsL*, *c-Jun*) via TLR2 signaling in mouse intestinal epithelial cancer cells [76]. Silver nanoparticles synthesized using EPS from *L. brevis* exhibited potential to act as dominant anti-proliferative agents against human cancer cell lines [77]. Ma et al. isolated two fengycin isoforms from marine *B. mojavensis* B0621A which were cytotoxic to human leukemia (HL-60) cells [78].

The α-synuclein (αSN) protein is associated with the Parkinson′s disease, and surfactants can modulate its structure. *P. aeruginosa*-derived monomeric rhamnolipid BS enhanced the capacity of αSN to permeabilize membranes, while micellar rhamnolipids induced the rapid transformation of protein β-sheet structure with a worm-like fibrillary appearance into linear fibrils. These data suggested that αSN aggregation and cell toxicity may be affected by interaction of rhamnolipids with αSN, which may implicate the propagation of Parkinson′s disease [79].

## 3. Application of Microbial Surfactants in Boosting the Immune System in Fish

Aquaculture is one of the fastest growing and most promising industries. Recently, several researchers have used bacterial secondary metabolites to control diseases in aquaculture species. We explored the immunomodulatory activities of BS extracted from *B. licheniformis* VS16 in fish [80]. *Labeo rohita* fingerlings were intraperitoneally (i.p) injected with various concentrations of the BS. Immunological parameters (e.g., lysozyme levels, alternative complement pathway (ACP), and phagocytic activities) and digestive enzymatic activities were higher (*p* < 0.05) in the BS-treated groups. We observed the down-regulation of *IL-1β, TNF-α, NF-κB p65*, and *IKK-β* and upregulation of *IL-10*, *TGF-β*, and *IKB-α* in the BS treated groups. BS dose of 220 µg/ mL was found to be optimal in inducing immune responses in fish and can be exploited for its immunomodulatory potential in fish. In a similar study, *B. subtilis* VSG4 derived BS at 200 µg mL^−1^ (i.p) positively influenced the immune responses (lysozyme, ACP, phagocyte, respiratory bursts, and serum bactericidal activity), resistance against *Aeromonas* challenge, and stimulated the expression of immune-related genes in *L. rohita* [81]. Another study demonstrated that lipopeptide BS isolated from halophilic *Bacillus* sp. BS3 was effective against shrimp white spot syndrome virus (WSSV) [58]. A biosurfactant isolated from a *Pseudomonas* strain had strong in vitro antiparasitic effect against the fish pathogenic ciliate *Ichthyophthirius multifiliis*. Further, effective doses (10 to 100 ug/mL) exhibited no adverse effects in rainbow trout fingerlings exposed to the biosurfactant, indicated the antiparasitic potential of the biosurfactant [82]. Phospholipopeptide BS isolated from *Staphylococcus hominis* was assessed for immunostimulatory activity in *Oreochromis mossambicus* [83]. Fish were injected i.p. with BS at the doses of 2, 20, or 200 mg kg^−1^ body weight. BS increased the immune responses and disease resistance of fish (*p* < 0.05). These results indicated that BS isolated from *S. hominis* could increase the aquaculture production by enhancing fish immunity.

Poly-3-hydroxybutyrate (PHB), synthesized by many bacteria, has been studied as a feed additive for several aquaculture species. WSSV causes heavy damage to the shrimp aquaculture. Monica et al. [84] studied the immunomodulatory role of PHB/BS against WSSV infection in shrimps. Increased levels of hemocytes were observed in both BS (28 ± 2 × 10^4^ cells)/PHB (26 ± 2 × 10⁴ cells) administered groups. Supplementation with 2% PHB or BS increased the survival rate of WSSV infected *P. monodon* which might be associated with observed over expression or down regulation of proteins involved in boosting the immune responses [84]. Various levels of dietary phospholipids or cholesterol strongly affected the expression of several immune-related genes in juvenile freshwater cultured *Litopenaeus vannamei* [85]. Moreover, dietary cholesterol and phospholipid supplementation resulted in better tolerance against *Vibrio alginolyticus* in shrimp.

In an interesting study, Laranja et al. demonstrated that a superior PHB-accumulating *Bacillus* strain JL47 improved the protective effects against a pathogenic *Vibrio campbellii* challenge in gnotobiotic *Artemia franciscana* [86]. Feng et al. investigated the effect of phospholipid (PL) supplementation on the immune responses and physical barrier of juvenile grass carp (*Ctenopharyngodon idella*) [87]. The dietary PL improved the antioxidant status, immune protection, and tight junction barrier of fish gills; thus, PL had a positive effect on the gill health. Based on alternative complement pathway (ACP) activity, complement C3, protein carbonyl content, and anti-superoxide anion activity in the gill, the optimum PL level for grass carp was estimated to be 3.62%, 4.30%, 3.91%, and 3.86%, respectively.

Recently, Qiao et al. conducted feeding trial with dietary PHB on soiny mullet (*Liza haematocheila*) [88]. Fish were fed for 60 days with basal diet containing 0, 0.05, 1, 2, 4, or 8% PHB at the rate, and various immunological and growth parameters were studied at 30- or 60-days post-feeding. Fish fed with 2% dietary PHB for 30 days had stronger antioxidant activity and expression of cytokine genes than 4% or 8% PHB supplementation groups. The stronger immune responses might be related to the changes in intestinal microbiota post-feeding of dietary PHB [88]. Similarly, Suguna et al. [89] investigated the immunostimulating potential of *Bacillus thuringiensis* B.t.A102-derived PHB-hydroxyvalerate (PHB-HV) [89]. They demonstrated that PHB-HV dietary supplementation was effective in stimulating both specific and nonspecific immune mechanisms in the fish *Oreochromis mossambicus*. Supplementation with 5% PHB-HV resulted in the highest post-challenge survival, then the other tested doses. Outcomes of these studies provided basic information on the regulation mechanism of PHB in aquatic animals.

The aquaculture employing biofloc technology (BFT) is gaining popularity presently. PHB is one of the most used compounds in biofloc [90]. PHB supplementation (at 4%) up-regulated (*p* < 0.05) the expression of cytokine genes and decreased the cumulative mortality of gibel carp. Moreover, PHB supplementation significantly increased the abundance of beneficial bacteria in the intestine. Hence, the authors suggested that the beneficial effects of PHB on fish immune responses might be related to the gut microbiota which regulates via the host immune system and related pathways [90].

The mammalian target of the rapamycin (mTOR) signaling pathway plays a vital role in intestinal inflammation and epithelial morphogenesis [91]. Dietary PHB supplementation increased the relative mRNA expression of TOR, 4E-BP, eIF4E1α, and eIF4E2, which are involved in mTOR signaling pathway in *L. vannamei* and improved the intestinal health by modulating microbial composition. PHB supplementation increased the disease resistance of nile tilapia larviculture against pathogenic *Edwardsiella ictaluri* [92]; thus, PHB may act as an antimicrobial agent in fish larviculture. Very recently, Sehgal Kiran et al. demonstrated the supplementation of 1% gelatinized PHB (isolated from *Brevibacterium casei* MSI04) effectively protected *P. vannamei* against *V. parahaemolyticus* infection, and improved the intestinal and immune enzyme activity [93]. It has been found that dietary supplementation with PHB-accumulating *Halomonas* strain provided better growth, survival, and disease resistance to *L. vannamei* than dietary supplementation with crystalline PHB [94].

Defoirdt et al. [95] investigated the mechanistic insight into the impact of PHB on the virulence trait of *V. campbellii* towards brine shrimp larvae. In the presence of 1000 mg L^−1^ PHB, 24 mM 3-hydroxybutyrate was detected in the intestinal tract of shrimp. This concentration of 3-hydroxybutyrate decreased the production of various virulence factors, including phospholipase, hemolysin, and protease without affecting the growth of *V. campbellii*. Further, through the challenge test it was confirmed that 3-hydroxybutyrate protected gnotobiotic brine shrimp from pathogenic *V. campbellii*, without affecting the number of host-associated vibrios. In another study, Laranja et al. revealed that feeding *Bacillus* sp. JL47 containing 55% amorphous PHB to *Artemia* resulted in significantly higher survival against the *Vibrio* challenge relative to the group fed with *Bacillus* containing 29% PHB [86]. These studies revealed that PHB is an important compound for the higher survival of shrimp or *Artemia* and could be exploited for its antimicrobial uses in fish aquaculture.

The outbreak of infectious diseases during larval rearing hampers the stable production of high-quality fry in marine aquaculture. Franke et al. investigated the effect of feeding PHB-enriched *Artemia nauplii* on the immune responses and survival of the European sea bass (*Dicentrarchus labrax*) post-larvae [96]. Although, the larval survival remained unaffected, the upregulated expression of the insulin-like growth factor 1 (*igf1*), an indicator of relative growth, was noticed. Ten days of PHB treatment elevated the expression of antimicrobial peptides hepcidin (*hep*) and dicentracin (*dic*), as well as *mhc class II a* and *mhc class II b* molecules in the PHB-fed postlarvae, indicating the immune stimulating capacity of PHB in the early life stages of fish [96]. Wang et al. [97] demonstrated that the addition of PHB as a feed additive increased the survival of large yellow croakers.

## 4. Conclusions and Future Implications

In the last decade, a tremendous interest in exploring the biological applications of microbial surfactants was noted. Currently, BS are considered an integral part in a wide array of industrial applications and offer a tremendous opportunity for more sustainable and biodegradable alternatives. Due to the immunosuppressive potential, few microbial surfactants are being investigated for their use in treating autoimmune diseases viz. asthma, diabetes, allergy, and arthritis. Applications of BS in immunology are still limited. Cost-effective and green production is vital for its wider biological applications. Using genetic modification of producer microorganisms, higher yield of BS is feasible.

Microbial surfactants, such as lipopeptides, can be exploited further for cancer treatment as they exhibit no or moderate toxicity against the normal lymphocytes. Many lipopeptides are being used as vaccine adjuvants to enhance the host’s immune response. Further research is warranted on recombinant lipopeptides for superior anti-cancer effects. Probiotic bacteria, especially of human origin, can be used for surfactant isolation, and such BS (lipopeptides) may not be rejected by our body and may have lesser side effects on the body.

Among glycolipids, trehalolipids from *Rhodococcus actinobacteria* are low in toxicity and have been found promising for applications in biomedicine. It has been reported that trehalolipids bind with receptors of the lectin family, resulting in high immunoregulatory activity. Decoding the mechanisms of trehalolipids and immune cells may pave the way for new therapeutic approaches [98].

Generally, biosurfactants are identified and classified based on their structure. However, there are few molecules for which the structure does not suggest a biosurfactant function, which is instead clearly based on phenotypic analyses. A recently discovered biosurfactant from *Myxococcus xanthus* might be an example of such a molecule. Thus, there might be many molecules with biosurfactant properties that are not yet classified because their structure does not suggest a biosurfactant function.

The therapeutic potential of probiotic bacteria-derived BS can be highly significant. The composition of many BS has not been fully elucidated. These BS molecules can be utilized in the prevention of hospital-acquired infections. The inhibition of microbial biofilm formation and the prevention of urogenital infection in mammals are important aspects in the utilization of BS. These aspects warrant more attention for the successful use of BS in diverse fields.

## Figures and Tables

**Table 1:** Major classes of microbial surfactants and their microbial source.

Type of Microbial Surfactants	Microbial Source
**Glycolipids**	
Rhamnolipids	*Pseudomonas aeruginosa*
Sophorolipids	*Candida apicola*, *C. bombicola*
Trehalose lipids	*Arthobacter* sp., *Rhodococcus erithropolis*
Mannosylerythritol lipids	*Candida antartica*
**Lipopeptides**	
Iturin/ surfactin/fengycin	*Bacillus subtilis*
Lichenysin	*Bacillus licheniformis*
Viscosin	*Psedomonas fluorescens*
Serrawettin	*Serrtia marcescens*
Phospholipids	*Acinetobacter* sp.
Fatty acids	
Corynomicolic acids	*Corynebacterium insidibasseosum*
**Polymeric surfactants**	
Alasan	*Acinetobacter radioresistens*
Emulsan	*Acinetobacter calcoaceticus*
Liposan/lipomanan	*Candida lipolytica*
**Particulate biosurfactants**	
Vesicles	*Acinetobacter calcoaceticus*
